# HNRNPK inhibits gastric cancer cell proliferation through p53/p21/CCND1 pathway

**DOI:** 10.18632/oncotarget.21873

**Published:** 2017-10-17

**Authors:** Hao Huang, Yong Han, Xingjiu Yang, Mengyuan Li, Ruimin Zhu, Juanjuan Hu, Xiaowei Zhang, Rongfei Wei, Kejuan Li, Ran Gao

**Affiliations:** ^1^ Key Laboratory of Human Disease Comparative Medicine, Ministry of Health, Institute of Laboratory Animal Science, Chinese Academy of Medical Sciences, Beijing 100021, P. R. China; ^2^ Department of Pathology, Zhejiang Provincial People's Hospital, Hangzhou 310014, Zhejiang, P. R. China; ^3^ People's Hospital of Hangzhou Medical College, Hangzhou 310014, Zhejiang, P. R. China; ^4^ Key Laboratory of Tumor Molecular Diagnosis and Individualized Medicine of Zhejiang Province, Hangzhou 310014, Zhejiang, P. R. China; ^5^ Department of Gynaecology and Obstetrics, Civil Aviation General Hospital, Beijing 100123, P. R. China

**Keywords:** gastric cancer (GC), HNRNPK, proliferation

## Abstract

Gastric cancer (GC) is one of the most common human cancers. The molecular mechanisms underlying GC carcinogenesis and progression are still not well understood. In this study, we showed that heterogeneous nuclear ribonucleoprotein K (HNRNPK) was an effective prognostic marker for GC patients especially in early stage. Overexpression of HNRNPK can retard tumor cell proliferation and colony formation *in vitro* and inhibit tumor growth *in vivo* through p53/p21/CCND1 axis. Bioinformatics analyses indicated that HNRNPK associated genes were enriched in cell cycle and DNA replication process. Protein-protein interaction network showed that HNRNPK was physically interacted with p53, p21 and other cancer related genes. Besides, GSEA showed that HNRNPK expression was positively correlated with GAMMA radiation response and DNA repair, while negatively correlated with angiogenesis, TGF-β and Hedgehog pathway activation. Finally, several chemicals including Glycine that may repress GC progression through upregulating HNRNPK are suggested. Our study demonstrated that HNRNPK may play as a tumor suppressor in gastric cancer and could be a potential therapeutic target for GC.

## INTRODUCTION

Gastric cancer (GC) is one of the most common cancers and the third cause in the global cancer-related mortality [1–3]. Early detection is the most promising approach to improve long-term survival of GC patients, but approximately 70% of GC patients are initially diagnosed in their late stages have missed the option of surgical resection [4]. Despite of improvements in cancer diagnosis and treatment, the prognosis of those GC patients is very poor with the 5-year survival rate less than 20% [5, 6]. Therefore, it is important to identify new prognostic markers and therapeutic targets to improve the detection of GC in early stage and targeted treatment.

Heterogeneous nuclear ribonucleoprotein K (HNRNPK), a component of the hnRNP complex, is a highly conserved RNA and DNA binding protein [7]. It contains three consecutive K homologue (KH) s, a nuclear localization signals (NLS) and a nuclear shuttling domain (KNS). KH domains are responsible for the binding of RNA or single stranded DNA, which consist of 65–70 amino acids [8]. NLS domain of HNRNPK serves upon its transport from the cytoplasm to the nucleus, and KNS domain promotes bi-directional translocating via the nuclear pore complex [9, 10]. HNRNPK preferentially and tenaciously binds to poly(C), and regulates transcription, translation, pre-mRNA splicing, RNA stability, chromatin remodeling, and signal transduction [11].

Recently, it has been reported that abnormal expression of HNRNPK is associated with tumor formation, development and prognosis. On one hand, cytoplasmic accumulation of HNRNPK in some tumors is associated with poor prognosis, overexpression of HNRNPK results in a potential oncogenic effect through regulation of c-Myc, c-Src and eIF4E [12–14]. On the other hand, HNRNPK acts as a transcriptional co-activator of p53; down-expression of HNRNPK attenuates p21 activation and C/EBP levels, and activates STAT3 signaling. Other than that, Hnrnpk haploinsufficient mice are tumor prone and develop malignant phenotypes, suggesting HNRNPK plays a potential role in tumor suppression [15–17]. Currently, the role of aberrant HNRNPK expression contributing to tumor phenotypes remains unclear.

In the present study, the prognostic value of HNRNPK expression in GC was investigated using a merged datasets provided by Dr. Györffy. The function and molecular mechanisms of HNRNPK in suppressing the proliferation of GC cells were explored through wet lab experiments and bioinformatics analyses.

## RESULTS

### HNRNPK was positively correlated with OS and FP in GC

**Figure 1:**
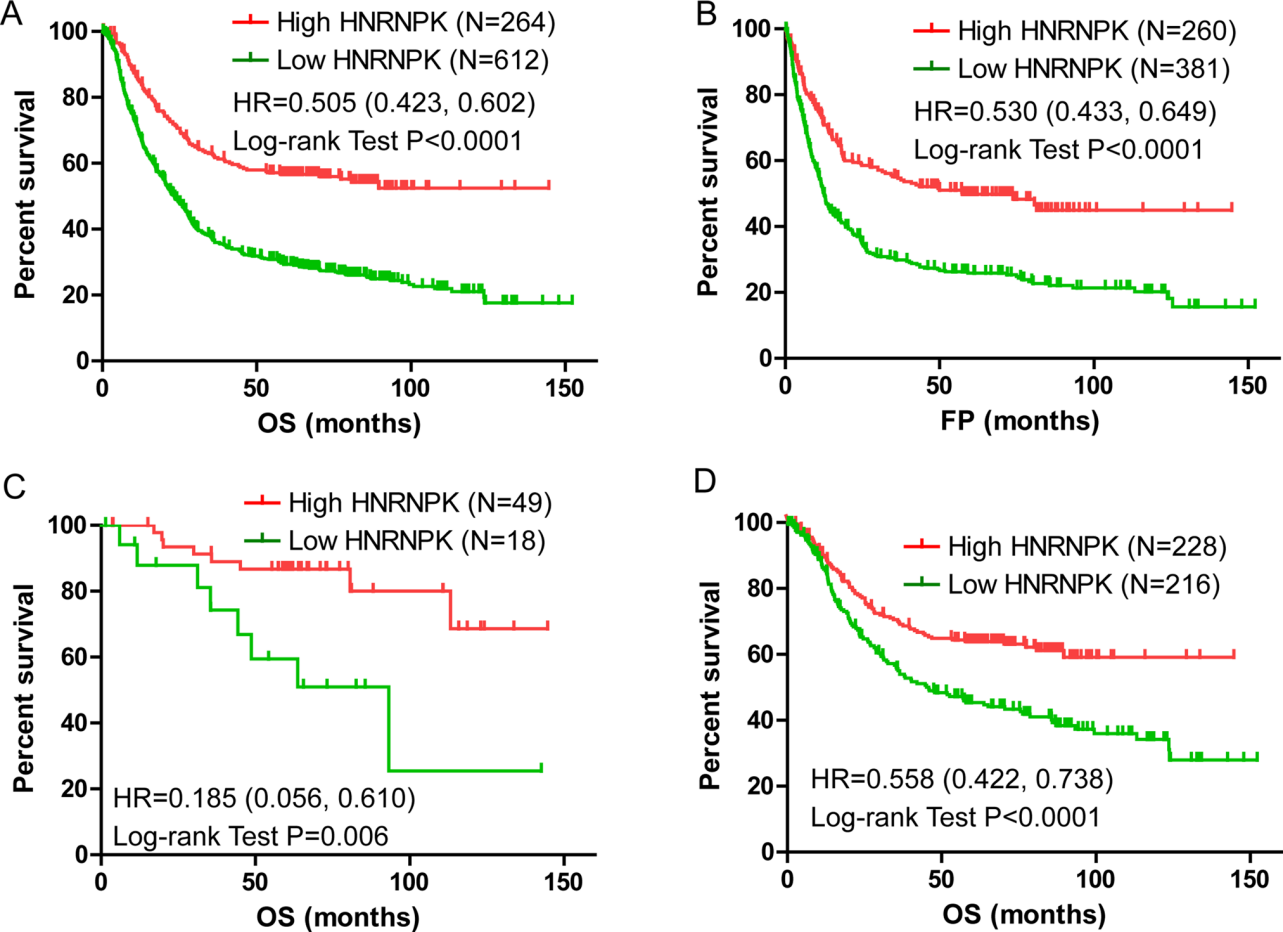
Kaplan-Meier analysis of OS and FP in GC patients HNRNPK expression was positively correlated with Overall Survival (OS) and Free of Progression survival (FP) in GC patients (**A** and **B**). HNRNPK expression was positively correlated with OS of GC patients with stage 1 (**C**) and no metastasis (M0) (**D**).

The gene expression datasets were kindly provided by Dr. Györffy. According to the mRNA transcript levels of HNRNPK, patients were classified into high and low level groups according to previously published criteria [18]. Kaplan–Meier analysis indicated that a low HNRNPK transcript level indicated poor overall survival (OS) and free of progression (FP) in GC patients (Figure 1A and 1B, *P* < 0.001 and *P* < 0.001). Further analysis showed HNRNPK expression was positively correlated with OS of GC patients with Stage 1 and no metastasis (M0) (Figure 1C and 1D, *P* = 0.006 and *P* < 0.001). These results indicated that a high level of the HNRNPK transcript could predict good outcomes for GC patients, and could be a candidate biomarker to evaluate the prognosis of patients in early stage.

### HNRNPK inhibited GC cell proliferation *in vitro*

**Figure 2:**
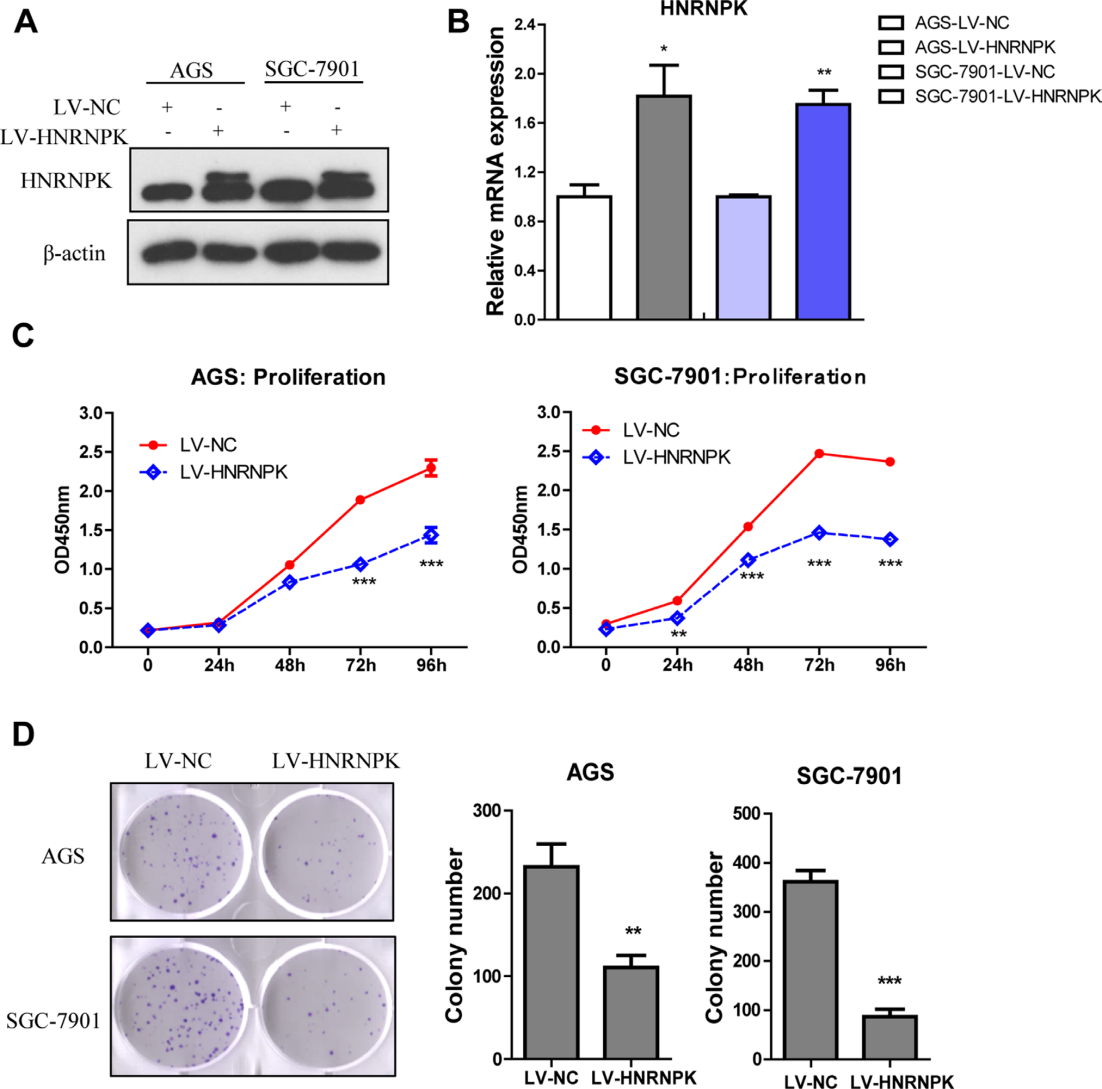
Effect of HNRNPK overexpression on the proliferation of GC cells *in vitro* HNRNPK was overexpressed using lentivirus (LV-HNRNPK, LV-NC as control ) in AGS and SGC-7901 cells, as detected by western blotting (**A**) and Real-Time PCR (**B**), cell proliferation as measured by CCK-8 assay (**C**), colony formation as stained with 0.5% crystal violet and the number of cell clones (**D**). The experiments were repeated three times independently. Error bars represent standard errors of the mean value. (^**^P < 0.01, ^***^P < 0.001)

To assess the efficiency of HNRNPK overexpression to cell proliferation, AGS and SGC-7901 cells were transduced with lentiviral vectors LV-HNRNPK and LV-NC. HNRNPK levels increased in LV-HNRNPK expressed cells, as shown by western blotting and Real-time PCR (Figure 2A and 2B). HNRNPK overexpression reduced AGS and SGC-7901 cell growth rates (Figure 2C, *P* < 0.001 at 72 h and 96 h ) and decreased the capacity of cell colony formation (Figure 2D , *P* < 0.01). Thus, HNRNPK overexpression inhibited GC cell proliferation *in vitro*.

### HNRNPK suppressed GC cell tumor growth in xenograft nude mice model (*In vivo*)

**Figure 3:**
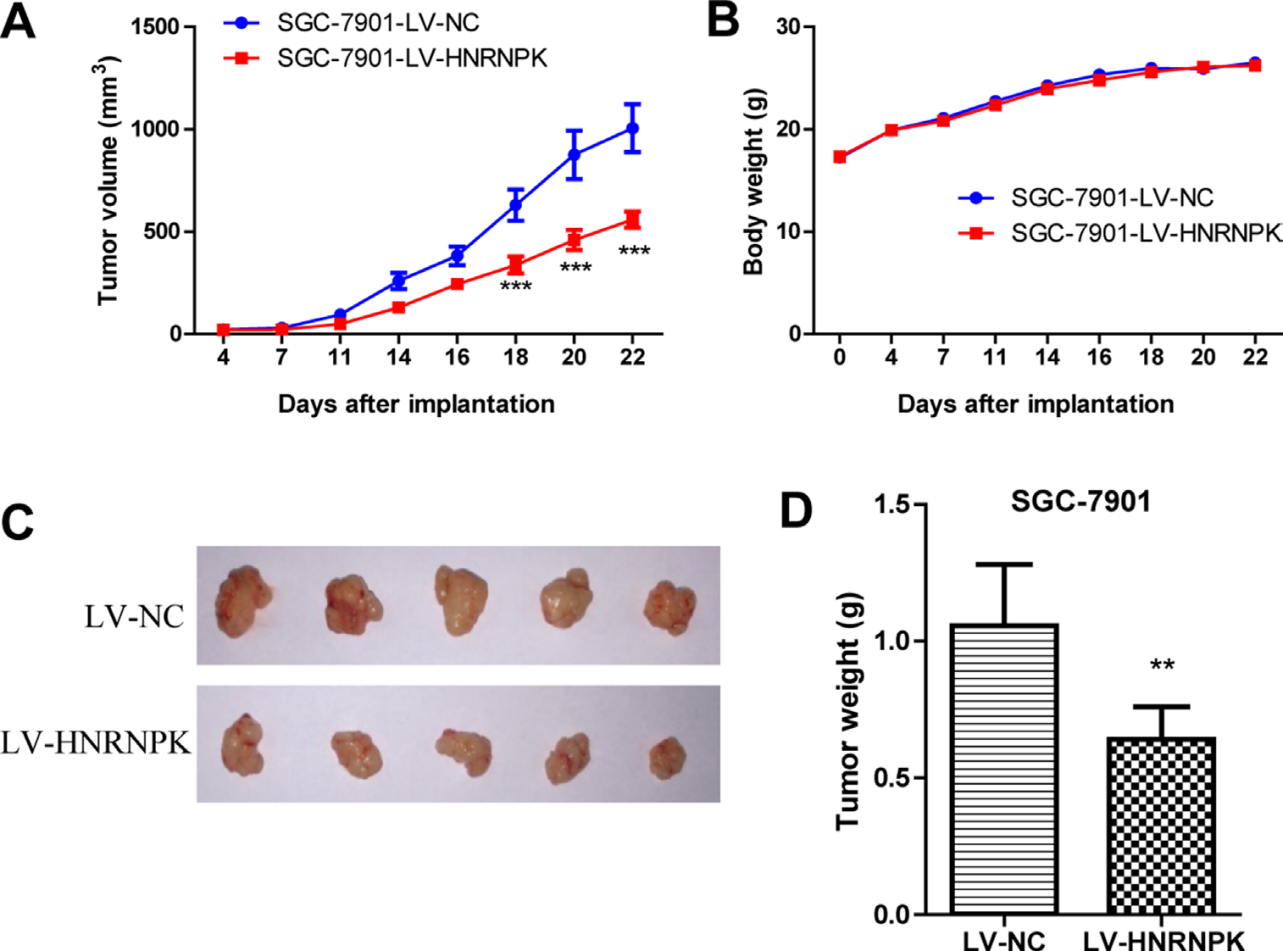
HNRNPK overexpression suppressed GC cell tumor growth *in vivo* The tumor growth volume (**A**) and body weight of mice (**B**) were measured twice a week, pictures of tumor xenografts by lentivirus infected SGC-7901 cells (**C**) and quantification of tumor weights (**D**). The results are presented as the means ± S.D. (*n* = 5). LV-NC as control. (^**^P < 0.01, ^***^P < 0.001).

**Figure 4:**
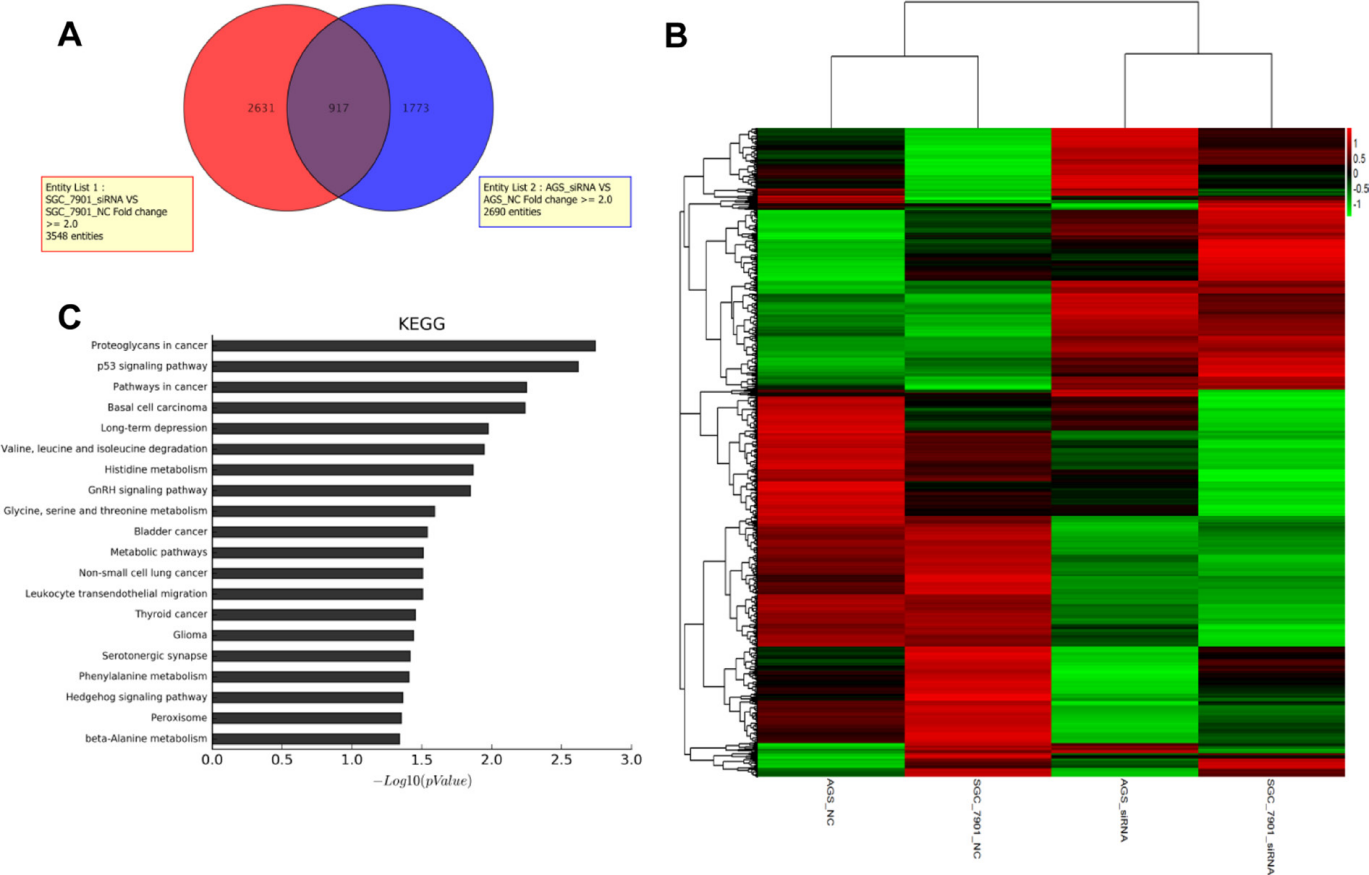
Function enrichment analysis of HNRNPK associated genes The Venn diagram showed the overlap between all genes that significantly change in HNRNPK knockdown group of AGS and SGC-7901 cells (**A**). The heatmap diagram showed the cluster of 917 overlap genes (**B**). The KEGG pathway analysis on the changed genes in HNRNPK knockdown group (**C**).

**Figure 5:**
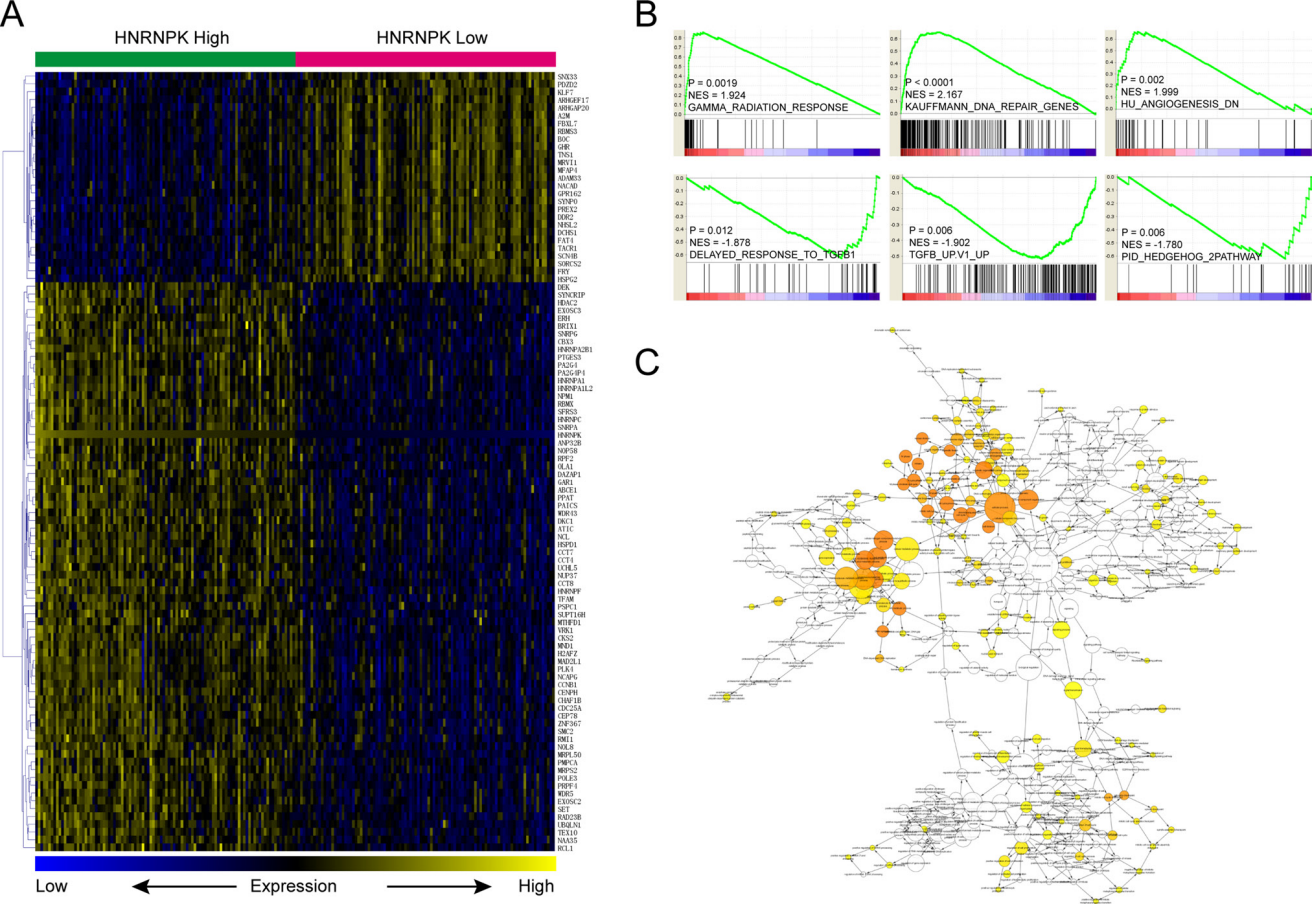
Molecular mechanisms underlie HNRNPK associated GC progression Cluster and heatmap visualization of top 100 differentially expressed genes between top and bottom 100 samples of gastric cancer ranked by HNRNPK (**A**). HNRNPK expression is positively correlated with GAMMA radiation response, DNA repair and signatures down-regulated in angiogenesis process, while negatively correlated with TGF-β and Hedgehog pathway activation (**B**). Function enrichment network of differentially expressed genes between top and bottom 100 samples of gastric cancer ranked by HNRNPK (**C**).

**Figure 6:**
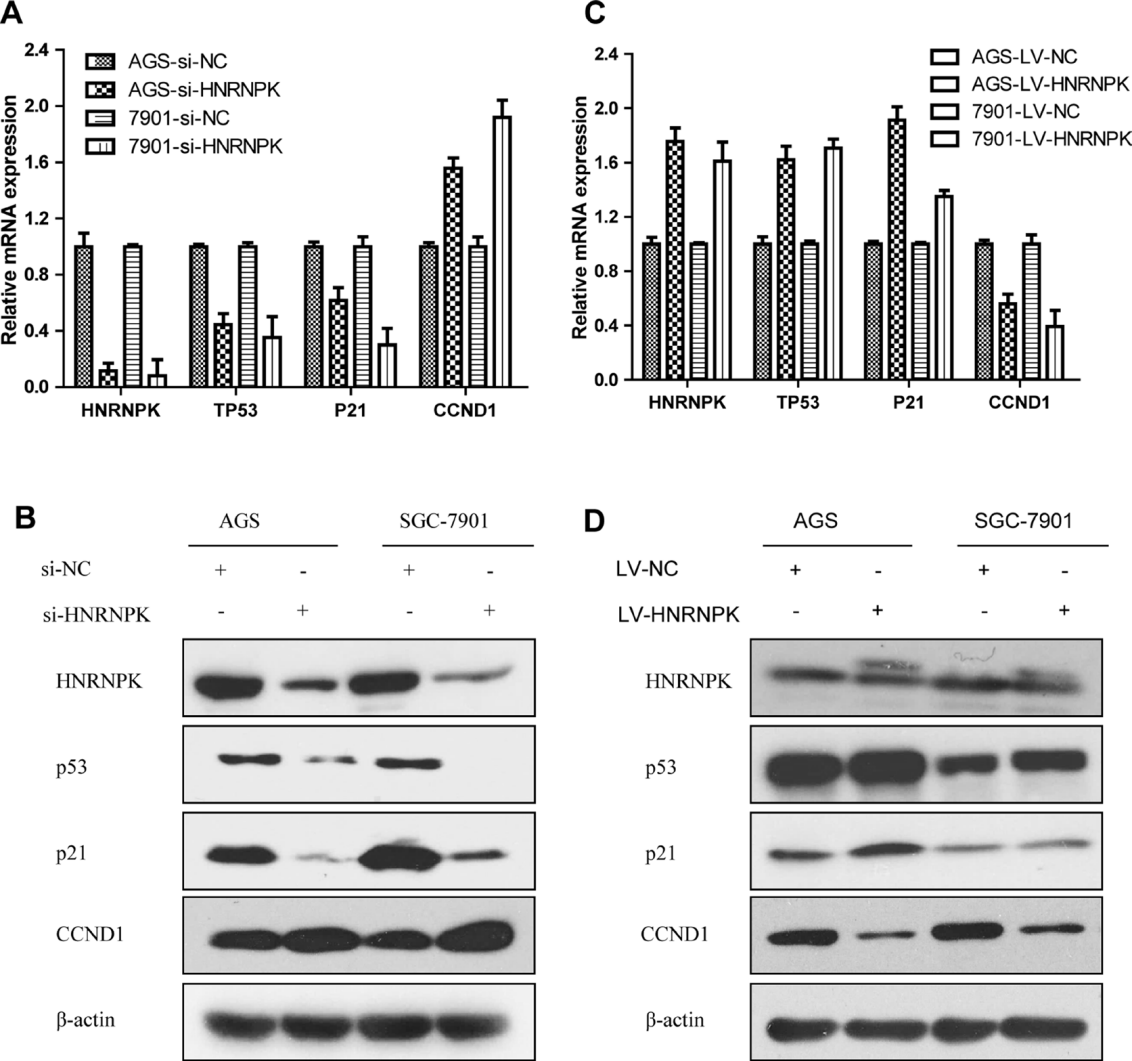
Effects of HNRNPK expression on p53 signaling pathway in GC cells After HNRNPK inhibition or upregulation using small interfering RNA or lentivirus in AGS and SGC-7901 cells, RNA and protein expression of p53, p21 and CCND1 were detected via quantitative real-time PCR (**A**, **C**) and western (**B**, **D**). p53, p21 were suppressed and CCND1 was upregulated in the HNRNPK small interfering RNA group, whereas overexpression of HNRNPK substantially upregulated p53, p21 and downregulated CCND1.

To explore the effect of HNRNPK on tumor growth *in vivo*, xenograft nude mouse model experiments were performed. The xenograft tumor growth in the LV-HNRNPK group (*n* = 5) were significantly slower than that in the negative control group (LV-NC), without difference in the body weight (Figures 3A–6D). These data suggested that HNRNPK overexpression suppressed GC cell tumor growth *in vivo*.

### Function enrichment analysis of HNRNPK associated genes

We investigated the downstream targets of HNRNPK by using mRNA microarray. Differential gene expression profiling analysis was performed using AGS and SGC-7901 cell lines transfected with HNRNPK small interfering RNA or negative controls. Compared with negative control group, 917 genes were significantly changed in HNRNPK-silenced gastric cancer cells (Figure 4A and 4B). These genes were enriched for gene ontology annotations and pathway analysis. The top three pathways selected were proteoglycans in cancer, p53 signaling pathway and pathways in cancer (Figure 4C), suggesting an important role of HNRNPK in cancer development.

Besides, we also downloaded and reanalyzed GC dataset from TCGA. Briefly, dataset was ranked by HNRNPK from high to low, and differentially expressed genes were computed by comparing top 100 samples with bottom 100 samples. The heatmap of top 100 differentially expressed genes were presented in Figure 5A (For details, please see Supplementary Table 1). GSEA results indicated that HNRNPK is positively correlated with GAMMA radiation response and DNA repair, while negatively correlated with angiogenesis, TGF-β and Hedgehog pathway activation (Figure 5B). Function enrichment analysis found that the differentially expressed genes were enriched in cell cycle and DNA replication etc. (Figure 5C, Supplementary Table 2).

### HNRNPK inhibited GC cell proliferation through p53/p21/CCND1 axis

For the sake of more intuitive experimental results, we next validated the effects of HNRNPK on the p53 pathway in AGS and SGC-7901 cells. Quantitative real-time PCR and western blotting confirmed that p53, p21 were suppressed and CCND1 was upregulated in the knockdown of HNRNPK group by transient transfection of small interfering RNA (Figure 6A and 6B), whereas overexpression of HNRNPK substantially upregulated p53, p21 and downregulated CCND1 (Figure 6C and 6D). Taken together, these data suggest that HNRNPK inhibits GC cell proliferation via the p53 signaling pathway, in which p53, p21 and CCND1 are involved.

### HNRNPK protein-protein & protein-drug interaction networks

List of HNRNPK physical interaction proteins were obtained from CTD base and visualized using GeneMANIA plugin in Cytoscape environment (Supplementary Figure 1, full list of physical interaction proteins are presented in Supplementary Table 3). This list demonstrated that p53 was proved to be physically interacted with HNRNPK by Affinity Capture-Western. p21 was validated to be interacted with HNRNPK by two-hybrid. Moreover, this protein-protein interaction network also indicated that HNRNPK was physically interacted with many other critical cancer associated genes such as EGFR, CD81 and AURKA.

Furthermore, by exploring CTD base, several ways to repress cancer progression through upregulating HNRNPK expression were suggested (Supplementary Figure 2). Cyclophosphamide, Dietary Fats, Glycine, SKF83959 and Sodium Selenite could upregulate HNRNPK and thus might mitigate GC progression.

## DISCUSSION

Over the past decade, *in vitro* and biochemical studies had shown that HNRNPK was involved in many cellular processes, which were crucial for tumorigenesis and cancer development [11]. HNRNPK overexpression and cytoplasmic accumulation in several tumors were associated with tumor progression and poor prognosis [19–21]. Abnormal cytoplasmic localization of HNRNPK was essential for cell invasion and metastasis [22–24]. These observations suggested that HNRNPK possessed oncogenic functions through overexpression and cytoplasmic functions. On the other hand, HNRNPK, as a transcriptional co-activator of p53, potentially functioned as a tumor suppressor and its loss also was prone to result in tumorigenesis [15, 16]. Mutations in HNRNPK were associated with driving AML progression, and deleted in AML patients [25, 26]. Hnrnpk haploinsufficient mice were tumor prone and developed malignant phenotypes; down-expression of HNRNPK directly attenuates attenuated p21 activation and influenced proliferation programs, suggesting that HNRNPK plays a potential role in tumor suppression [17]. Taken together, the role of aberrant HNRNPK expression in tumor formation and development remains unclear because of multiple cellular processes it regulates.

In this study, we analyzed the relationship between transcript levels of HNRNPK and patients’ clinical outcomes in GC. Herein, we demonstrated that HNRNPK expression was positively correlated with OS of GC patients in early stage, suggested HNRNPK transcript level as a candidate biomarker to evaluate the prognosis of GC patients in early stage. Uncontrolled cell proliferation is a major factor in cancer development and progression [27, 28]. Overexpression of HNRNPK in GC cell lines displayed a significant reduction in proliferation, colony formation *in vitro* and tumor growth *in vivo*. These results indicated that HNRNPK plays a tumor suppressor and influences proliferation activities in GC.

To uncover the potential molecular mechanisms of regulation of HNRNPK on cell proliferation in GC, gene expression microarray was performed. KEGG pathway analysis revealed that differentially expressed genes between group of HNRNPK knockdown and negative control were enriched in the pathways of proteoglycans in cancer, p53 signaling and cancer, suggesting an important role of HNRNPK in cancer development. Knockdown of HNRNPK in GC cells blocked the p53 signaling pathway, and reduced the expression level of p53 and p21, while HNRNPK upregulation produced the opposite results. The p53 signaling pathway regulates various cell functions, including the induction of apoptosis and senescence, and the inhibition of cell growth, migration, and invasion [29]. p53 could induce G1/S and G2/M cell cycle arrest via cell cycle checkpoints [30, 31]. As a target gene of p53, p21 also plays an important role in cell growth, cell cycle, arrest and invasion [32].

Cyclin D1, a key regulator in G1-to-S-phase transition, is overexpressed and/or amplified in a large fraction of human cancers [33, 34]. Clinic pathological studies demonstrated that cyclin D1 overexpression correlated with tumor metastasis and poor prognosis in a series of human cancers [35]. Previous studies have showed that cyclin D1 allows progression from G1 to S phase by binding and sequestering p21 [36]. Ectopic expression of miR-326, miR-134, miR-329, and miR-206 in NSCLC cell lines significantly suppressed cell proliferation through inhibition of cyclin D1 and up-regulation p21 [37–40]. In response to DNA damage and other stresses, HNRNPK, as a cofactor of p53 and a substrate of MDM2, the level of which and p53 increased and was recruited to the promoters of p53-reponsive genes, including p21 and HDM2 [15]. Gallardo et al. demonstrated that HNRNPK was required and indeed a direct regulator for activation of p21 in a p53-dependent manner following DNA damage [17]. These data suggested that HNRNPK regulates the proliferation of GC cells mainly by regulation of p53, p21 and cyclin D1.

Bioinformatics analyses using GC data of TCGA confirmed that HNRNPK associated genes were enriched in cell cycle and DNA replication process. CTD base datamining indicated that p53 and p21 were physically interacted with HNRNPK. These results were in accordance with our findings. Besides, high radiation response, efficient DNA repair, low angiogenesis ability, inactivation of TGF-β and Hedgehog pathway may also account for HNRNPK related good outcomes. Moreover, several potential options such as Cyclophosphamide, Glycine and Sodium Selenite to upregulate HNRNPK expression were suggested. Interestingly, Glycine can be acquired from natural food sources and could be used to lower symptoms of ulcers, arthritis, diabetes, kidney and heart failure, chronic fatigue and sleep disorders. If validated, Glycine would be a promising therapeutic option for HNRNPK low expression subgroup of GC patients.

Based on current knowledge, it is still difficult to define HNRNPK as an oncogene or a tumor suppressor gene because of dichotomous results from clinical association data and cell line studies. The role of HNRNPK in tumor development and progression may depend on the tissue type and/or binding partners. Hnrnpk+/− mouse model has been proved tumor suppressive function of HNRNPK, however, transgenetic mouse model that overexpress HNRNPK will still be critical for determining the role of HNRNPK, whether it has oncogenic potential.

In summary, it is our novel discovery that low transcript level of HNRNPK correlates with poor prognosis in gastric cancer, especially in early stage and no metastasis patients. Moreover, HNRNPK overexpression inhibits gastric cancer cell proliferation and tumor growth by regulating p53 signaling pathway suggesting that HNRNPK may play as a tumor suppressor in gastric cancer and could be a potential therapeutic target.

## MATERIALS AND METHODS

### Cell lines and cultures

GC cell lines AGS and SGC-7901 were obtained from the Institute of Basic Medical Sciences, Chinese Academy of Medical Sciences and cultured in RPMI-1640 medium (Gibco, USA) containing 10% fetal bovine serum (Gibco, USA), 100 U/mL penicillin, and 100 mg/mL streptomycin (Invitrogen, Grand Island, NY, USA) in a humidified atmosphere of 5% CO_2_ at 37°C.

### Cell transfection

Overexpressed HNRNPK related lentivirus vector (LV-HNRNPK) and negative control vector (LC-NC) were purchased from Shanghai Gene Chem Co.Ltd. (Shanghai, China). Cells at 30%–50% confluence were transfected with lentivirus on six-well plates in accordance with the manufacturer’s recommendations. Cells were continuously cultured for one week to select the clones stably overexpressing HNRNPK. Small interfering RNA (siRNA) against HNRNPK (si-HNRNPK) and non-specific control siRNA (si-NC) were purchased from Ribo (Guangzhou, China). The target sequence of siRNA-HNRNPK was 5′-GAGCUUCGAUCAAAAUUGATT and si-NC was 5′-UUCUCCGAACGUGUCACGUTT. All cells were transfected by Lipofectamine 2000 (Invitrogen) and harvested after 48 h for further studies.

### Western blotting

Cells were homogenized in RIPA lysis buffer (Promega, USA) on ice for 30 min. Protein concentrations were estimated using the BCA protein assay (Pierce). Equal amounts of cell lysates were loaded and separated on 12% SDS-PAGE gels, and the proteins were electrotransferred to nitrocellulose membranes, as described previously [41]. The membranes were incubated with 1:1000 dilution of anti-HNRNPK, anti-p53, anti-p21, anti-CCND1 antibodies (Cell Signal Technology, USA) and anti-β-actin (1:10000 dilution, Santa Cruz, CA) antibody overnight at 4°C in 5% nonfat milk, followed by washing three times with TBS-T buffer and then incubated with a horseradish peroxidase-conjugated goat anti-rabbit IgG (1:10000 dilution with 5% nonfat milk) for 45min at room temperature. After washing three times with TBS-T buffer, the membranes were visualized with ECL detection system (Thermo, USA).

### Microarray analysis and quantitative real-time PCR assay

Total RNAs were extracted from cultured cells with Trizol reagent (Invitrogen) following the manufacture’s protocol. Gene expression profiles were examined by ShangHai OE Biotechnology Corporation (Shanghai, China) using Agilent SurePrint G3 Human Gene Expression v2 (8 × 60K, Design ID: 039494) microarrays. After robust multi-array average normalization, fold change threshold ≥ 2 and *P* value < 0.05 were identified to be statistically significant alterations. Hierarchical clustering and heat map generation were performed using GeneSpring GX software (Agilent Technologies). Signaling pathways that enriched in downstream target genes were generated using the KEGG pathways program (http://www.kegg.jp/).

Complementary DNA (cDNA) was synthesized with ImProm-IITM Reverse Transcription System (Promega) from 1 μg of RNA. Quantitative PCR was performed using ABI StepOne Real-Time PCR system (Applied Biosystems) and SYBR Green Realtime PCR Master Mix (Toyobo). Primers used for quantitative real-time PCR are listed in Table 1. All reactions were carried out in triplicate and the relative gene expression was calculated using the comparative cycle threshold (2^-ΔΔCt^) method following the manufacturer’s instructions. The house-keeping gene glyceraldehyde-3-phosphate dehydrogenase (GAPDH) was used as the endogenous control to normalize the data. Primers were synthesized by Sangon Biotech (Shanghai, China).

**Table 1:** Primer sequence

Gene name		Primer sequence (5′ to 3′)
HNRNPK	Forward	AGACCTGGAGACCGTTAC
	Reverse	ATAAGCCATCTGCCATTC
TP53	Forward	CCAGGGCAGCTACGGTTTC
	Reverse	CTCCGTCATGTGCTGTGACTG
P21	Forward	TGAGCCGCGACTGTGATG
	Reverse	GTCTCGGTGACAAAGTCGAAGTT
CCND1	Forward	ACAAACAGATCATCCGCAAACAC
	Reverse	TGTTGGGGCTCCTCAGGTTC
GAPDH	Forward	CATCAAGAAGGTGGTGAAGCAG
	Reverse	CGTCAAAGGTGGAGGAGTGG

### Cell proliferation assay

CCK8 kits (Dojindo, Tokyo, Japan) were used to evaluate cell proliferation of cells. Cells were seeded into 96-well plates at a density of 2 × 10^3^ cells per well, followed by the addition of CCK8 reagents at different time points, then incubated for an additional 3 h at 37°C, each concentration was set in triplicate. The absorbance was measured using a microplate reader (Bio-Rad, USA) at a wavelength of 450 nm (OD450nm) after slight oscillation for 10 s.

### Colony formation assay

Transfected gastric cancer cells were seeded into 6-well plates at 1000 cells/well, and incubated at 37°C with 5% CO_2_ for ten days. Surviving colonies (> 50 cells/colony) were stained with 0.5% crystal violet and counted.

### Tumor xenograft model

Animal experiments were approved by the Biomedical Ethical Committee of Chinese Academy of Medical Sciences (GR-16002). Male BALB/c nude mice aged 6 to 8 weeks (Beijing Huafukang Biotechnology) were housed under specific pathogen-free conditions. Gastric cancer cells respectively infected with lentivirus expressing HNRNPK (LV-HNRNPK) and control (LV-NC) were injected into subcutaneous in the right flank of each mouse. The tumor length (L) and width (W) were measured every 3 days, 3 weeks later; all mice were sacrificed and solid tumors were harvested, weighed, and pictured. Tumor volume = (W^2^L)/2.

### Bioinformatics and statistical analysis

GC data from The Cancer Genome Atlas (TCGA) was download for computing HNRNPK related genes which were used for Gene Set Enrichment Analysis (GSEA) and BiNGO analysis. HNRNPK physical interaction proteins and potential drugs that could upregulate HNRNPK were obtained from CTDbase. Network visualization was performed using GeneMANIA plugin in Cytoscape environment.

Statistical analysis was performed using GraphPad Prism 5.01 software. All values were expressed as means ± SD and representative of at least 3 independent experiments. *P* < 0.05 was defined as statistical significance.

## SUPPLEMENTARY MATERIALS FIGURES AND TABLES









## Abbreviations

HNRNPK: heterogeneous nuclear ribonucleoprotein K
GC: gastric cancer
TCGA: The Cancer Genome Atlas
GSEA: Gene Set Enrichment Analysis
OS: overall survival
FP: free of progression
KH: K homologue
NLS: nuclear localization signal
CCK8: cell counting kit-8
CTDbase: The Comparative Toxicogenomics Database
