# Supplementary material for: HNRNPK inhibits gastric cancer cell proliferation through p53/p21/CCND1 pathway
Source: Oncotarget. 2017 Oct 17;8(61):103364–74. doi: 10.18632/oncotarget.21873 (PMC5732733; doi:10.18632/oncotarget.21873)
Supplement: Supplementary file 1 [file oncotarget-08-103364-s001.pdf]

HNRNPK inhibits gastric cancer cell proliferation through p53/p21/CCND1 pathway

SUPPLEMENTARY MATERIALS

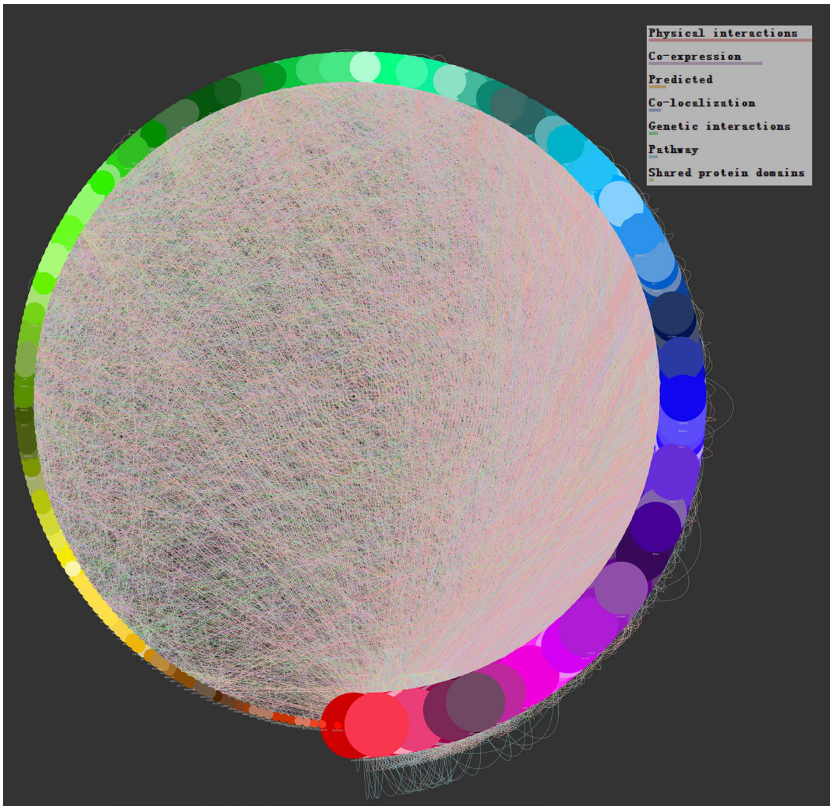

Supplementary Figure 1: Protein-protein interaction network of HNRNPK.

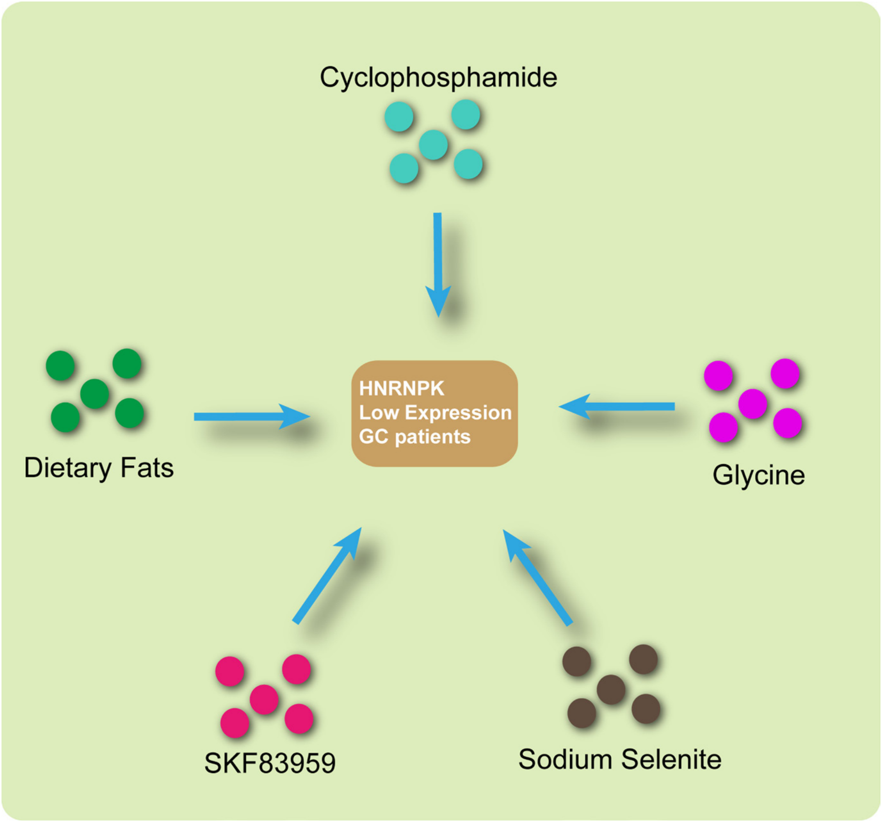

Supplementary Figure 2: Chemicals that can upregulate HNRNPK.

Supplementary Table 1: Differentially expressed genes between top 100 samples and bottom 100 samples of TCGA gastric cancer expression data which ranked by HNRNPK expression levels. See Supplementary_Table_1

Supplementary Table 2: Gene annotation analysis of differentially expressed genes by GeneMANIA plugin in cytoscape environment. See Supplementary_Table_2

Supplementary Table 3: Physical interaction proteins with HNRNPK. See Supplementary_Table_3
